# Around the Hospitals

**Published:** 1892-09-24

**Authors:** 


### AROUND THE HOSPITALS.

**The Sheffield Infirmary** has just held its ninety-fifth annual meeting in connection with a building erected so far back as 1797. It is not surprising that large sums have to be expended over structural repairs, and that, therefore, a large deficiency in the accounts stares the management in the face. An entirely new drainage scheme, not effected one minute before it was necessary, has absorbed £1,615; £370 has been spent in restoring windows, and £350 on new lifts. These are really solid and necessary items, such as fairly might be the subject of a special appeal. The infirmary requires its ordinary income for the every day expenses of a useful work. Two hundred beds are available for patients, 40 of which are reserved for children; and 1,637 cases have been received during the past year. At the meeting it was decided to elect the Dukes of Devonshire and Portland to be Vice-Presidents of the Infirmary.

**The Sunderland Infirmary** is an example of those institutions which show a steady increase in useful work done and general prosperity. We do not mean by this that the coffers of the institution are as full as they might be, but that, as indications of the necessity of enlarging the sphere of work of the infirmary have arisen, funds have been forthcoming. Ten years ago 6,534 in-patients were received; now, in 1892, no less than 16,849 have been accommodated. The working classes have helped greatly in this excellent work of a decade by generous contributions, which, during that time, have amounted to £19,047. The latest addition to the infirmary is the new isolation block, just opened by Lady Londonderry, the gift of Miss Foster, of South Hetton. The building, which was erected at a cost of £3,000, is very handsome externally and well-arranged and fitted within. Previous additions to the institution during the past ten years have been: (1) The Backhouse Wing; (2) public room, boiler houses, and offices; (3) out-patients' department; (4) the Hartley Wing. The infirmary system is rendered further complete by the opening of the Heath Dene Convalescent Home, which the governors have been enabled to secure by the aid of the Victoria Hall Calamity Fund. The Home will add to the expenditure of the institution to the extent, it is calculated, of £1,000 a year. Doubtless friends will respond to the laudable desire of the management to render the Sunderland Infirmary excellent in every respect by further generous assistance.

**The Salisbury Infirmary** has been undergoing many alterations and additions of late, both as to structure and constitution, so that it may be said to have commenced a new term of life. It has just passed its 126th anniversary. The number of beds is now raised to 118; some years ago the beds were reduced from 120 to 108. Although the accommodation has been again extended, financially the institution will not be in a condition to support the increase, unless further subscriptions are received. At the present moment stock is being sold for the purpose of paying for necessary items, and to reduce the debt to the treasurer. In the Matron, the Committee find able assistance in keeping down unnecessary expenditure, and during her management expenses have been considerably reduced. She has also effected considerable reforms in the nursing department. The nursing students, of whom two are admitted at the same time on payment of £5 for a term of six months, are an especial and useful feature. We regret to see that a proposal to advance the Matron's salary to £90 was not acted upon. It is a matter of policy alone to encourage evident zeal, and to make every effort to keep possession of it, for the good of the institution. The latest improvements in the infirmary have been those effected in Accident Bartlett, the Ovariotomy Ward, and the new dispensary. It is now intended to erect a clock tower for the City of Salisbury, on a portion of the infirmary grounds, for which purpose the Town Council have purchased 20 feet of ground from the institution. The style of the clock tower is to accord with the masonry of the infirmary, and will therefore add to the general appearance of it.

